# A first-principles study of the effect of vacancy defects on the electronic structures of greigite (Fe_3_S_4_)

**DOI:** 10.1038/s41598-018-29176-1

**Published:** 2018-07-30

**Authors:** Min Wu, Xia Zhou, Shibei Huang, Jianlin Cheng, Zhenyu Ding

**Affiliations:** 1 College of Materials Science and Engineering, Zhejiang University of Technology, Hangzhou, 310014 P. R. China; 2 College of Mechanical Engineering, Zhejiang University of Technology, Hangzhou, 310014 P. R. China

## Abstract

Greigite (Fe_3_S_4_) is a ferrimagnetic mineral with an inverse spinel structure. Besides its importance in the bio-geochemical cycle, it also has great potential applications for its unique properties such as its half metallic electronic structure at ambient condition. However, it has been challenging to get high purity and crystallinity samples of greigite in experiment, and the defect effect on the electronic structure of greigite was not clear. In the present study, first-principles calculations have been performed to investigate the ground state electronic structure of greigite with monovacancy. It is found that, with an vacancy concentration lower than 3.6%, the greigite with an Fe vacancy is an insulator with charge orderings, while the greigite with a S vacancy becomes a half-metal and has a magnetic moment of <4.0 μB per formula unit. The present result helps to understand the absence of the Verwey transition and the magnetic property of greigite measured in experiment. The understanding of the electronic structure of defective greigite could also be utilized to manipulate the properties of greigite for spintronic applications.

## Introduction

Greigite (Fe_3_S_4_) was first discovered as a mineral by Skinner *et al*. from Californian lake sediments^1^, and its natural form was probably from the bacteriological reduction of iron^2^. It was recognized that greigite is an intermediate state in the solid-state transformation pathway from mackinawite (FeS) to pyrite (FeS_2_)^3^. Greigite is an important mineral for geophysics and biology, since it records the ancient geomagnetic field and environmental processes which are crucial for paleomagnetic and environmental magnetic studies^4–7^. On the other hand, the unique properties of greigite also have attracted extensive studies. For instance, greigite is considered to have potential spintronic application for its half metallic electronic structure at ambient condition with a high curie temperature^8^. It has also been suggested to be useful as an anode material in lithium-ion batteries^9^. Furthermore, the inductive heating property of greigite nanoparticle makes it a candidate for cancer hyperthermia application^10^.

Greigite is a sulfide counterpart of the well-known magnetite (Fe_3_O_4_)^11^. It has an inverse spinel structure with one tetrahedral iron site and two octahedral iron sites per formula unit (Fe^3+^_A_(Fe^2+^_B_Fe^3+^_B_)S^2−^_4_). Here the subscripts A and B represent the tetrahedral and octahedral cation site, respectively. In our previous work, first-principles calculations with hybrid functional method showed that the insulative monoclinic structure of greigite induced by charge ordering is energetically more stable than the half-metallic cubic phase, indicating the Verwey transition may exist in greigite^12^. However, the Verwey transition has not been observed in greigite in experiment so far. On the other hand, the magnetic moment of greigite measured in experiment was smaller than 4.0 μB per formula unit which is expected for the fully ionic model^2,9,13–17^. It was suggested that the covalence bonding feature participated in greigite was the main reason that lowered the saturated magnetic moment^14,16^. It should be noticed that the metastable nature and the challenges in obtaining high-quality samples of greigite could also be one of the reasons for the absence of the Verwey transition and its relatively low measured magnetic moment. The purity and crystallinity of greigite samples have been proved to have strong effect on the magnetic properties. For instance, the initial magnetic moment of greigite was reported to be ~2.2 μB per formula unit^2^ but was increased dramatically to ~3.7 μB with an improved sample^9^. Although the neutron diffraction results indicate no significant vacancy concentration that departures from the stoichiometry in the high quality synthetic greigite^7^, it is still worth investigating the ground state electronic structures and magnetic properties of defective greigites with reasonable defect concentrations. General density functional theory (DFT) using the generalized gradient approximation (GGA) had been proven to result in an underestimated lattice constant and an incorrect electronic structure of greigite^12^. The previous calculations using the Hubbard U method showed the electronic structure of greigite is highly sensitive to the effective U value^18^. Hybrid functionals by mixing a portion of the exact nonlocal exchange of Hartree–Fock theory usually provide more reliable description of the electronic structure of highly correlated systems, but the computation is very demanding. In the present study, first-principles calculations using the Heyd-Scuseria-Ernzerhof screened hybrid functional (HSE)^19^ and Hubbard U methods were performed to investigate the ground state electronic structure and magnetic properties of greigite with monovacancy. The presented results show the defects have strong effects on the electronic structure of greigite and help to understand the properties of greigite measured in experiment.

## Results and Discussion

**Figure 1:**
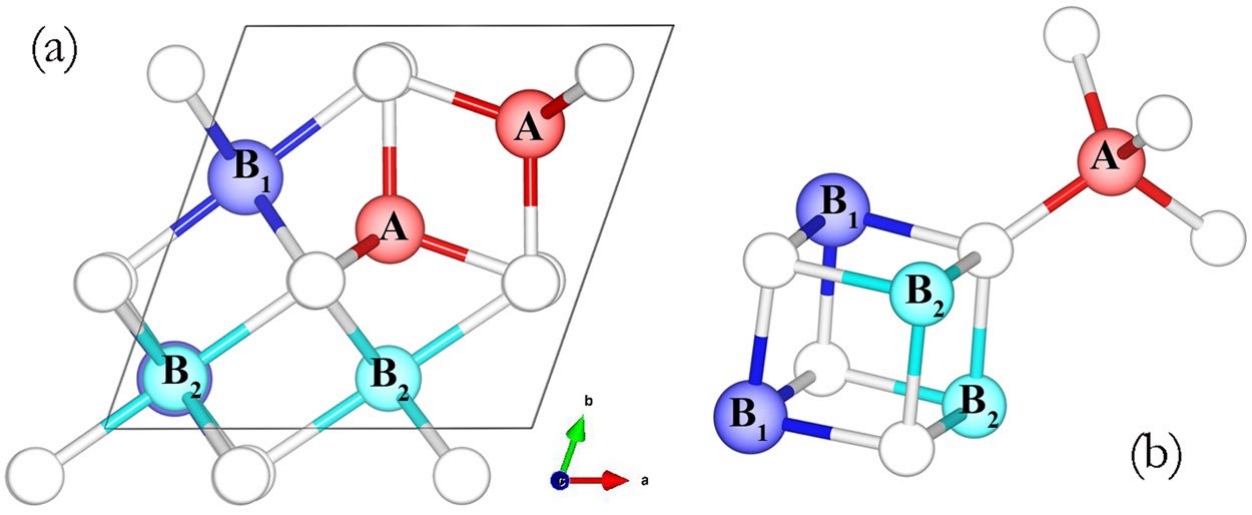
(**a**) The primitive cell of greigite. The red, blue, light blue and white balls are the tetrahedral Fe_A_ atoms, octahedral Fe_B1_ atoms, Octahedral Fe_B2_ atoms and S atoms, respectively. (**b**) Illustration of the local structure of greigite.

HSE calculation on a primitive cell of greigite with monovacancy was performed and used as the benchmark for the subsequent Hubbard U calculation. The primitive cell containing 14 atoms with 2 formula units was used as the starting structural model for further vacancy creation. (Fig. 1). In the initial spin configuration, the tetrahedral Fe_A_ sites have the opposite spin against the octahedral Fe_B_ sites. Anderson condition was used for the charge ordering, the same as our previous work^12^. In order to minimize the finite-size effect induced by the image-charge interaction^20^, Hubbard U calculations with larger cell containing 28 and 56 atoms have also been performed. To determine the U value for the large cell calculation, calculations on the primitive cell using a series of U values (U = 1, 2, 3, 4, 5 eV) were performed to compare with the HSE results. Three different vacancy sites including the tetrahedral Fe_A_ site, the Octahedral Fe_B_ site and the S site were considered in the present study.

It should be mentioned that general DFT calculations with PBE functional on the three defective models all result in metallic states, in which the Fe 3*d* orbitals are delocalized and dominates the Fermi level. The unrealistic itinerant Fe 3*d* state is similar to the result from the general DFT calculation on the pristine Fe_3_S_4_. On the other hand, hybrid functional and Hubbard U method are more appropriate for such highly correlated systems. In our previous work, the calculation with a U value larger than 3 eV results in a more stable monoclinic structure of geigite which is the same as the result from HSE calculation^12^. Electronic density of states (DOS) of the defective primitive cell using the HSE method and GGA + U method with a series of U values were compared in Fig. S1 (see supplementary information). Similar to our previous work on the pristine greigite^12^, the GGA + U calculation with a U value of 4 eV shows the best agreement with the HSE result. This value is very close to Liu *et al.’*s work on the electronic calculation of magnetite (Fe_3_O_4_) where an effective U value of 3.61 eV was used^21^. Thus the U value of 4 eV will be used for larger cell calculation which is too demanding for the HSE method. The vacancy formation energy, structural information and the magnetic moment from HSE calculation on a primitive cell and GGA + U calculation (U = 4 eV) on three different unit cell sizes are listed in Table 1. The structural models with vacancy at Fe_A_, Fe_B_ and S sites are hereafter labelled as Fe_A_-vac, Fe_B_-vac and S-vac, repectively. The vacancy formation energy E_vac_ was calculated from equation

1 $${{\rm{E}}}_{{\rm{form}}}={{\rm{E}}}_{{\rm{vac}}}+{{\rm{U}}}_{{\rm{atom}}}-{{\rm{E}}}_{{\rm{pristine}}}$$

where E_form_ is the vacancy formation energy, E_vac_ is the total energy of the greigite structure with monovacancy, U_atom_ is the chemical potential of the vacancy atom in its bulk form (S atom in S_8_ and Fe atom in bulk Fe), E_pristine_ is the total energy of the pristine greigite.

**Table 1:** Data of the pristine Fe_3_S_4_ and the defective Fe_3_S_4_ with monovacancies.

		E_form_ (eV)	V (Å^3^)	a (Å)	b(Å)	c (Å)	M_s_ (μB)
Exp.			120.41				
HSE (14-atoms cell)	Pristine		127.23	7.118	7.118	7.092	4.00
	FeA-vac	3.41	122.58	7.025	7.025	7.019	7.01
	FeB-vac	3.64	122.52	7.043	7.043	7.004	2.94
	S-vac	0.55	133.07	7.245	7.245	7.239	3.83
GGA + U (14-atoms cell)	Pristine		129.91	7.169	7.169	7.130	4.00
	FeA-vac	3.26	126.01	7.090	7.090	7.089	7.00
	FeB-vac	3.60	125.12	7.099	7.099	7.047	2.81
	S-vac	0.50	136.77	7.326	7.326	7.234	4.00
GGA + U (28-atoms cell)	FeA-vac	3.38	129.05	7.159	7.147	10.088	5.99
	FeB-vac	3.47	127.92	7.135	7.117	10.076	3.50
	S-vac	0.71	131.78	7.222	7.260	10.053	3.49
GGA + U (56-atoms cell)	FeA-vac	3.40	129.84	10.129	10.133	10.120	5.00
	FeB-vac	3.80	129.26	10.117	10.110	10.110	3.74
	S-vac	0.91	131.06	10.150	10.160	10.167	3.74

E_form_ is the vacancy formation energy; V is the volume of the cell; a,b and c are the lattice parameters; M_s_ is the averaged magnetic moment per Fe_3_S_4_ unit. The units of distance, volume, angle and magnetic moment are in Å, Å^3^, degree and μB, respectively.

### HSE calculations

The HSE calculation shows that the volume of the Fe_A_-vac structure shrank by 3.7% compared to the pristine structure. The formation of the Fe_A_ vacancy enhances the bonding between the S atoms near the vacancy and its nearest Fe_B_ atoms. Consequently, the averaged Fe_B_-S distance decreased from 2.477 Å in the pristine structure to 2.444 Å. The magnetic moment per formula unit cell is 7.0 μB, much larger than the predicted value of the pristine structure which is 4.0 μB. The increased magnetic moment with an Fe_A_ vacancy is expected since the minor Fe_A_ sites have opposite spins against the Fe_B_ sites.

For the structure with an Fe_B_ site vacancy, the volume shrank by 3.7% compared to the pristine structure and is very close to the Fe_A_-vac structure. The creation of the Fe_B_ site vacancy leaves unsaturated electrons on the nearest S atoms, which consequently causes stronger bonding between these S atoms and their nearest Fe atoms. As a result, the Fe_A_-S distance decreased from 2.265 Å to 2.240 Å and Fe_B_-S distance decreased from 2.477 Å to 2.429 Å, respectively. The magnetic moment per formula unit is 2.94 μB, which is much smaller than the pristine structure. The decreased magnetic moment is expected because the defective Fe_B_ is the major site contributing to the magnetic moment.

In both the Fe_A_-vac and Fe_B_-vac structures, the cell volume decreased with the introduction of the Fe vacancies. However, the reduced number of S atoms which are anions in the S-vac structure will lower the oxidation states of the Fe atoms and weaken the Fe-S bonding. As a result, the volume was increased by 4.6% compared to the pristine structure, the averaged Fe_A_-S distance increased from 2.265 Å to 2.284 Å.

From the comparison of the vacancy formation energy, it shows that the S vacancy requires much less formation energy than the Fe vacancy and should be the common type if there is any vacancy existing in the greigite sample. However, the vacancy concentration in the primitive cell is too high to be realistic. Larger unit cells with smaller vacancy concentrations are needed for further investigations.

### GGA + U calculations

From the comparison of the total density of state (DOS) as shown in Fig. S1 and the data listed in Table 1, the results from the GGA + U calculations with the U value of 4 eV agree well with the HSE calculation in the primitive cell. The GGA + U calculations with larger unit cells were also performed and compared to the result in the primative cell (Table 1). From the comparison of the vacancy formation energies, it is clear that the S vacancy always requires a much less formation energy (at least 2.5 eV less) compared to the Fe vacancy in all three different sized cells. In the structural point of view, the influence of a vacancy should be smaller in a larger unit cell with a diluted vacancy concentration. For example, as discussed above, the Fe_A_ vacancy will induce shrinkage of the volume. The reduced volume percentages for 14, 28 and 56-atoms cells are 3.0%, 0.7% and 0.1%, respectively. Such trend of diluted reduced volume percentage in larger unit cells can also be found in the result of Fe_B_-vac structure. On the other hand, the volume expansion induced by the S vacancy was found in all three different sized cells, indicating the expansion was indeed induced by the local structural distortion near the vacancy, rather than the image-charge interaction because of the finite cell size. The expanded volume percentage for 14, 28 and 56-atoms cells are 5.3%, 1.4% and 0.9%, respectively.

**Figure 2:**
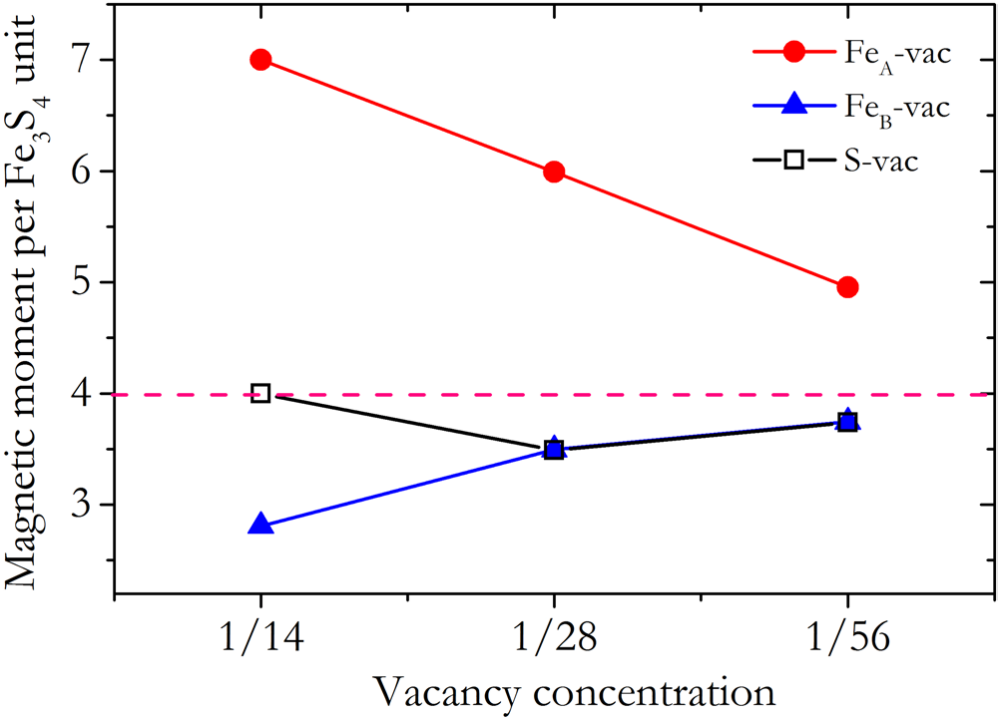
The vacancy concentration dependent magnetic moment per Fe_3_S_4_ formula unit. The red, blue and black symbols represent the results of Fe_A_-vac, Fe_B_-vac and S-vac structures, respectively. The solid lines are the guides for eyes.

The vacancy concentration dependent magnetic moment per Fe_3_S_4_ formula unit was shown in Fig. 2. It can be found that only the Fe_A_ vacancy will increase the total magnetic moment. While for the Fe_B_ and S vacancies, they both led to reduced total magnetic moments compared to the pristine greigite structure when the vacancy concentration is smaller than 3.6% (1/28). Since the S vacancy should be the common vacancy defect in the greigite sample according to the comparison of the vacancy formation energy, it would not be surprising that the defective greigite has a magnet moment smaller than 4.0 μB per formula unit.

**Figure 3:**
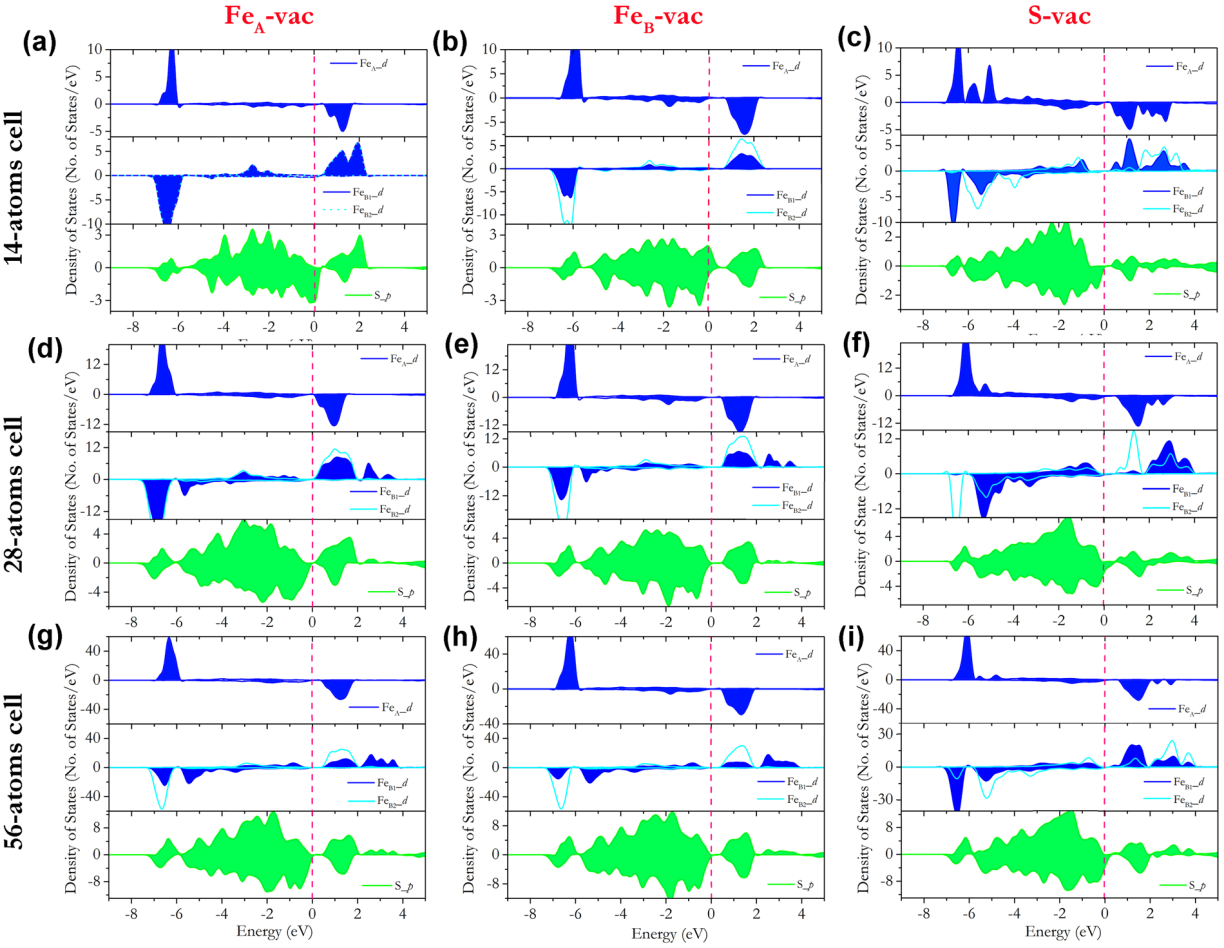
PDOS of the defective greigite structures in different sized cells. (**a**) Fe_A_-vac in 14-atoms cell; (**b**) Fe_B_-vac in 14-atoms cell; (**c**) S-vac in 14-atoms cell; (**d**) Fe_A_-vac in 28-atoms cell; (**e**) Fe_B_-vac in 28-atoms cell; (**f**) S-vac in 28-atoms cell; (**g**) Fe_A_-vac in 56-atoms cell; (**h**) Fe_B_-vac in 56-atoms cell; (**i**) S-vac in 56-atoms cel. The Fermi level is adjusted to 0 eV and marked as the red dashed line. The PDOSs are the summation of the atomic PDOS of each atom type.

To further investigate the electronic structures of greigite with these three monovacancies, the vacancy concentration dependent DOS and projected density of states (PDOS) were shown in Fig. 3. It should be mentioned that, in our previous work on the pristine greigite structure, the existence of the charge ordering induced the metallic-to-insulator transition. Figure 3(a,b,c) shows the PDOS of the defective greigite in the 14-atoms primitive cell. With such a high vacancy concentration (7.1%), no charge ordering was found in the Fe_A_-vac and Fe_B_-vac structures and resulted in a half-metallic state in the former and a metallic state in the later. The Fe_B__3*d* orbitals in Fe_A_-vac and Fe_B_-vac structures mainly located at −6.5 eV and −6.0 eV, with no orbital energy difference between Fe_B1_ and Fe_B2_ atoms. From the bader atomic charge comparison between the Fe_B1_ and Fe_B2_ atoms as shown in Table 2, the bader charges of Fe_B1_ and Fe_B2_ in the Fe_A_-vac and Fe_B_-vac structures with 14-atom primitive cell are very close, indicating the absence of the charge ordering. On the other hand, the charge ordering was found in the S-vac structure and led to an insulative state. The Fe___3*d* orbital energies of Fe_B1_ and Fe_B2_ differed by about 1.5 eV. However, as suggested above, calculations using a larger unit cell are needed to minimize the finite-size effect and avoid the image-charge interaction for a more realistic description of the electronic structure of defective greigite.

**Table 2:** Bader atomic charge comparison between Fe_B1_ and Fe_B2_ in greigite with different vacancies and vacancy concentrations.

		eB1	eB2	Δ*e*
GGA + U (14-atoms cell)	FeA-vac	6.814	6.830	0.016
	FeB-vac	6.859	6.858	0.001
	S-vac	6.891	6.953	0.062
GGA + U (28-atoms cell)	FeA-vac	6.873	6.844	0.029
	FeB-vac	6.883	6.837	0.046
	S-vac	6.931	6.895	0.036
GGA + U (56-atoms cell)	FeA-vac	6.907	6.842	0.065
	FeB-vac	6.956	6.863	0.093
	S-vac	6.906	6.952	0.045

Δ*e* is the bader charge difference between Fe_B1_ and Fe_B2_ atoms.

In the calculations of the 28-atoms cell (Fig. 3(e,d,f)), the charge orderings appeared in both Fe_A_-vac and Fe_B_-vac structures, which is in sharp contrast to the results in the 14-atomss cell. The main distribution of the Fe_B1__3*d* orbitals split into two peaks and located at −6.5 eV and −5.5 eV. The bader charge differences between Fe_B1_ and Fe_B2_ increased to 0.029 e and 0.046 e in the Fe_A_-vac and Fe_B_-vac structures, respectively. The occurrence of the charge ordering opened the energy gap and led to insulative states in the Fe_A_-vac and Fe_B_-vac structures. On the other hand, the charge ordering still existed in the S-vac structure. Interestingly, the charge ordering only opened the energy gap in the Fe_3*d* orbitals while electronic distribution at Fermi level was found in the S_*p* orbitals which led to a half-metallic state.

The PDOS from the calculations of the 56-atoms cell were shown in Fig. 3(g,h,i). The result is similar to that of the calculation in the 28-atoms cell. The Fe_A_-vac and Fe_B_-vac structures were still insulator with charge ordering in the Fe_B_ atoms, while the S-vac structure remained half-metallic. With the further diluted vacancy concentration, the nature of the pristine greigite structure became stronger. As shown in Fig. 3(g,h), the distribution of the Fe_B1__3*d* electrons at −5.5 eV in both Fe_A_-vac and Fe_B_-vac structure increased compared to that of calculations in the 28-atoms cell, indicating a stronger charge ordering. This is consistent with the bader charge results. With further diluted vacancy concentration in the 56-atoms cell, the bader charge differences between Fe_B1_ and Fe_B2_ further increased to 0.065 e and 0.093 e in the Fe_A_-vac and Fe_B_-vac structures, respectively.

From the results presented above, it indicates that the very different electronic structures of the defective greigites in a 14- atoms cell and a 28-atoms cell could be due to the finite-size effect induced by the image-charge interaction. Such finite-size effect was significantly minimized by using a large 58-atoms cell which shown converged results with respect to the results using the 28-atoms cell.

## Conclusions

The electronic structures of greigite (Fe_3_S_4_) with three types of vacancies (Fe_A_-vac, Fe_B_-vac and S-vac) have been studied by the first-principles calculations. From the comparison of the vacancy formation energy, it was found that the creation of a S vacancy requires much less energy than the Fe vacancy at either the Fe_A_ site or the Fe_B_ site. It indicates the S vacancy should be the common vacancy if there is any existing in the defective greigite sample in experiment. With a vacancy concentration smaller than 3.6%, the S-vac structure has a magnetic moment less than 4.0 μB per Fe_3_S_4_ formula unit. This result could help to understand the unsaturated magnetic moment of greigite measured in the experiment, since it is difficult to synthesise high purity and high crystallinity greigite sample and the existence of defects such as the S vacancy is not surprising. On the other hand, the finite-size effect was significantly minimized by using larger cells of 28 and 56 atoms. It shows that the charge ordering can be found in all the defective structures, except the structures in a 14-atoms cell which has a relatively unrealistic vacancy concentration. The charge ordering led to insulative states for both Fe_A_-vac and Fe_B_-vac structure. However, the charge ordering in the S-vac structure only opened the energy gap in the Fe_3*d* orbitals. Conductive spin-down electrons were found in the S_*p* orbitals which led the S-vac structure to a half-metallic state. This result may also explain the experiment result that no Verwey transition has been observed in greigite so far. The present theoretical results not only help to understand the experimentally measured properties of greigite which may contain vacancies, but also will trigger more experimental and theoretical studies to understand or even manipulate the properties of greigite by introducing selected types of defects.

## Method

All the calculations were performed using the Vienna Ab initio Simulation Package (VASP)^22^. The projector augmented wave (PAW) potential^23^ with the generalized gradient Perdew, Burke, and Ernzerhof (PBE)^24^ exchange-correlation density function was employed. The Valence configurations of 3*d*^6^4*s*^2^ and 3*s*^2^3*p*^4^ were used for the Fe and S atoms, respectively. Plane-wave was expanded with an energy cutoff of 400 eV. The *k*-point convergence was tested until the energy difference is smaller than 1 meV/atom. All calculations were performed in the spin-unrestricted method. The cell and atoms are fully optimized until the Hellmann-Feynman forces on all the atoms are less than 0.01 eV/Å. The energy convergence criterion for the electronic self-consistent calculation was 10^−4^ eV.

## Electronic supplementary material

Supplementary Information

## References

[1] Skinner BJ, Erd RC, Grimaldi FS (1964). The thiospinel of iron; a new mineral. Am. Mineral..

[2] Spender MR, Coey JMD, Morris AH (1972). The Magnetic Properties and Mossbauer Spectra of Synthetic Samples of Fe_3_S_4_. Can. J. Phys..

[3] Hunger S, Benning LG (2007). Greigite: a true intermediate on the polysulfide pathway to pyrite. Geochem. Trans..

[4] Snowball IF, Thompson R (1988). The occurrence of greigite in sediments from Loch Lomond. J. Quat. Sci..

[5] Snowball IF, Thompson R (1990). A stable chemical remanence in Holocene sediments. J. Geophys. Res..

[6] Ron H (2007). Greigite detected as dominating remanence carrier in late Pleistocene sediments, Lisan Formation, from Lake Kinneret (Sea of Galilee), Israel. Geophys. J. Int..

[7] Roberts AP (2006). Characterization of hematite (a-Fe_2_O_3_), goethite (a-FeOOH), greigite (Fe_3_S_4_), and pyrrhotite (Fe_7_S_8_) using first-order reversal curve diagrams. J. Geophys. Res..

[8] Wang J, Cao SH, Wu W, Zhao GM (2011). The Curie temperature and magnetic exchange energy in half-metallic greigite Fe_3_S_4_. Phys. Scr..

[9] Li GW (2014). High-Purity Fe_3_S_4_ greigite microcrystals for magnetic and electrochemical performance. Chem. Mater..

[10] Chang YS, Savitha S, Sadhasivam S, Hsu CK, Lin FH (2011). Fabrication, characterization, and application of greigite nanoparticles for cancer hyperthermia. Journal of Colloid and Interface Science..

[11] Verwey EJW, Haayman PW, Romeijn FC (1947). Physical Properties and Cation Arrangement of Oxides with Spinel Structures II. Electronic Conductivity. J. Chem. Phys..

[12] Wu M, Tse JS, Pan YM (2016). Electronic structures of greigite (Fe_3_S_4_): A hybrid study and prediction for a Verwey transition. Sci Rep..

[13] Aragon R (1992). Magnetization and exchange in nonstoichiometric magnetite. Phys. Rev. B.

[14] Chang L (2008). Fundamental magnetic parameters from pure synthetic greigite (Fe_3_S_4_). J. Geophys. Res..

[15] Braga M, Lie SK, Taft CA, Lester WA (1988). Electronic structure, hyperfine interactions, and magnetic properties for iron octahedral sulfides. Phys. Rev..

[16] Roberts AP, Chang L, Rowan CJ, Horng C‐S, Florindo F (2011). Magnetic properties of sedimentary greigite (Fe3S4): An update. Rev. Geophys..

[17] Chang L (2009). Magnetic structure of greigite (Fe_3_S_4_) probed by neutron powder diffraction and polarized neutron diffraction. J. Geophys. Res..

[18] Devey AJ, Grau-Crespo R, de Leeuw NH (2009). Electronic and magnetic structure of Fe_3_S_4_: GGA + U investigation. Phys. Rev. B.

[19] Heyd J, Scuseria GE, Ernzerhof M (2003). Hybrid functionals based on a screened Coulomb potential. J. Chem. Phys..

[20] Freysoldt C, Neugebauer J (2009). Fully *Ab Initio* finite-size corrections for charged-defect supercell calculations. Phys. Rev. Lett..

[21] Liu X, Yin L, Mi WB (2017). Biaxial strain effect induced electronic structure alternation and trimeron recombination in Fe_3_O_4_. Sci Rep..

[22] Kresse G, Hafner J (1994). Norm-conserving & ultrasoft pseudopotentials for first-row and transition elements. J. Phys. Condens. Matter.

[23] Bloechl PE (1994). Projector augmented-wave method. Phys. Rev. B.

[24] Perdew JP, Burke K, Ernzerhof M (1996). Generalized gradient approximation made simple. Phys. Rev. Lett..

