# Supplementary material for: A first-principles study of the effect of vacancy defects on the electronic structures of greigite (Fe3S4)
Source: Sci Rep. 2018 Jul 30;8:11408. doi: 10.1038/s41598-018-29176-1 (PMC6065442; doi:10.1038/s41598-018-29176-1)
Supplement: Supplementary file 1 — Supplementary Information [file 41598_2018_29176_MOESM1_ESM.doc]

**Supporting information**

A first-principles study of the effect of vacancy defects on the electronic structures of greigite (Fe_3_S_4_)

Min Wu^1^, Xia Zhou^1^, Shibei Huang^1^, Jianlin Cheng^1^, Zhenyu Ding^2,*^

^1^College of Materials Science and Engineering, Zhejiang University of Technology, Hangzhou 310014, P. R. China

^2^College of Mechanical Engineering, Zhejiang University of Technology, Hangzhou 310014, P. R. China

Corresponding author: [*zyding@zjut.edu.cn](mailto:*zyding@zjut.edu.cn)


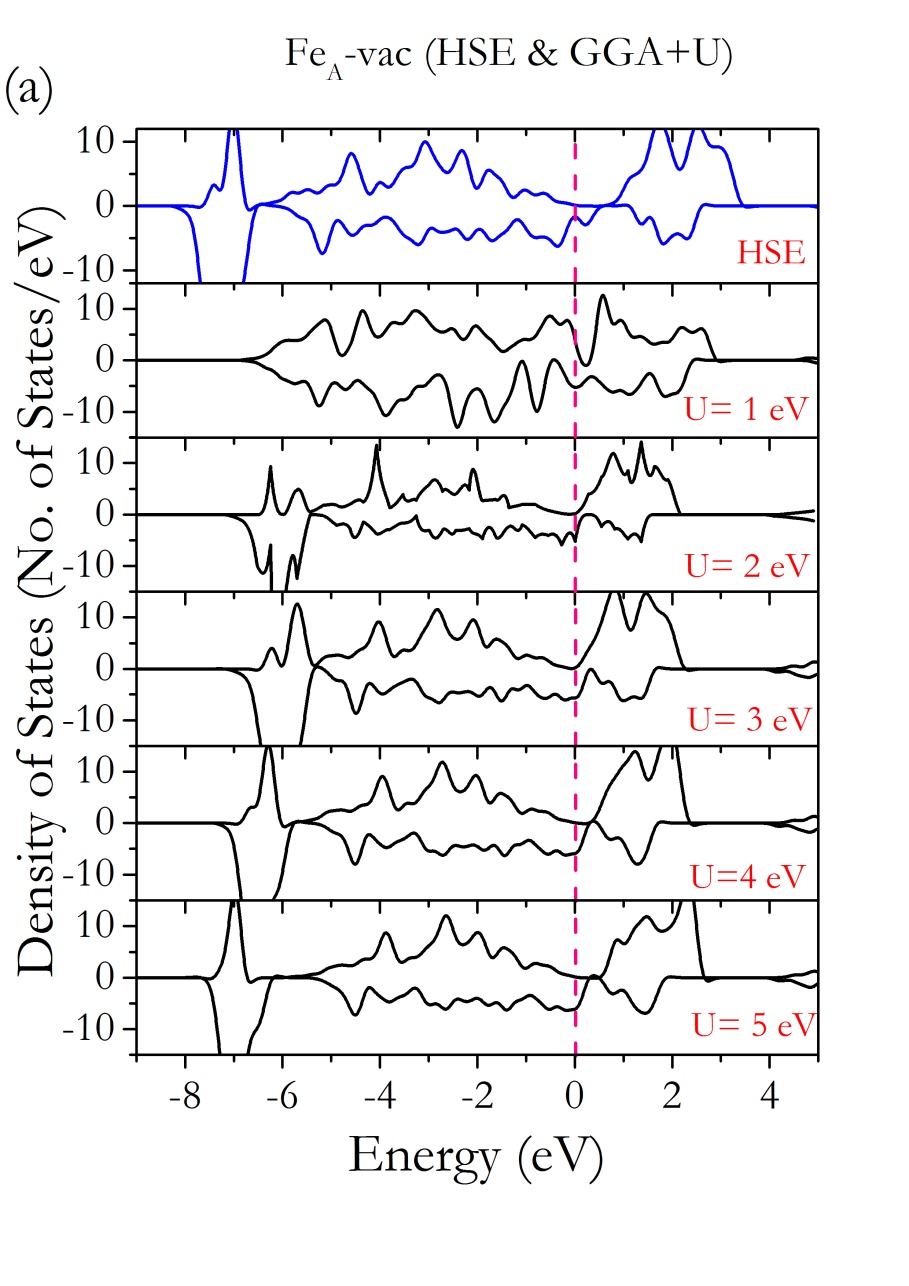


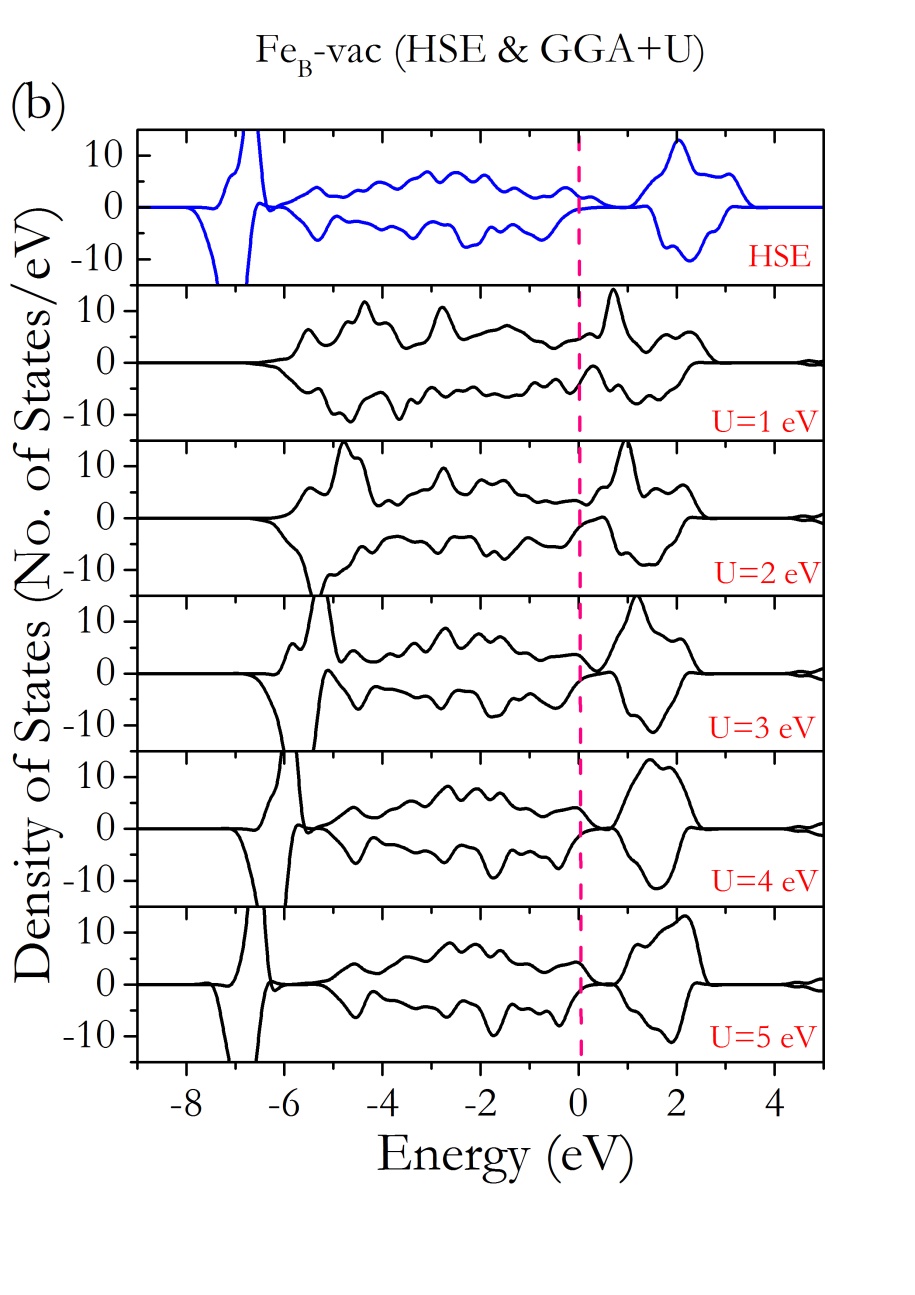


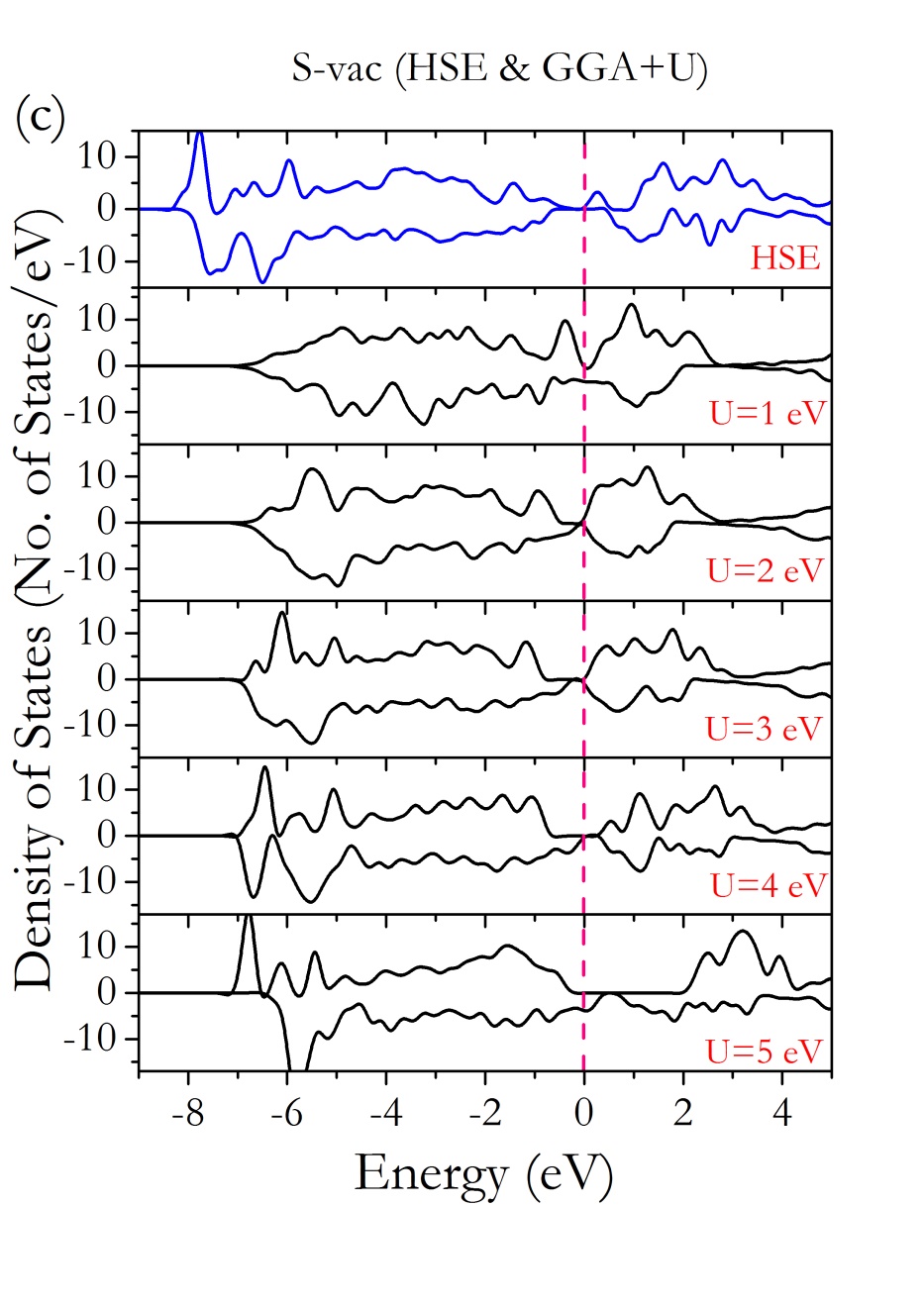


Fig. S1 Comparison of the electronic density of states of defective greigite in primitive cell calculated from HSE and GGA+U methods. (a) DOS of greigite with monovacancy at the tetrahedral Fe site. (b) DOS of greigite with monovacancy at the octahedral Fe site. (c) DOS of greigite with monovacancy at the S site.


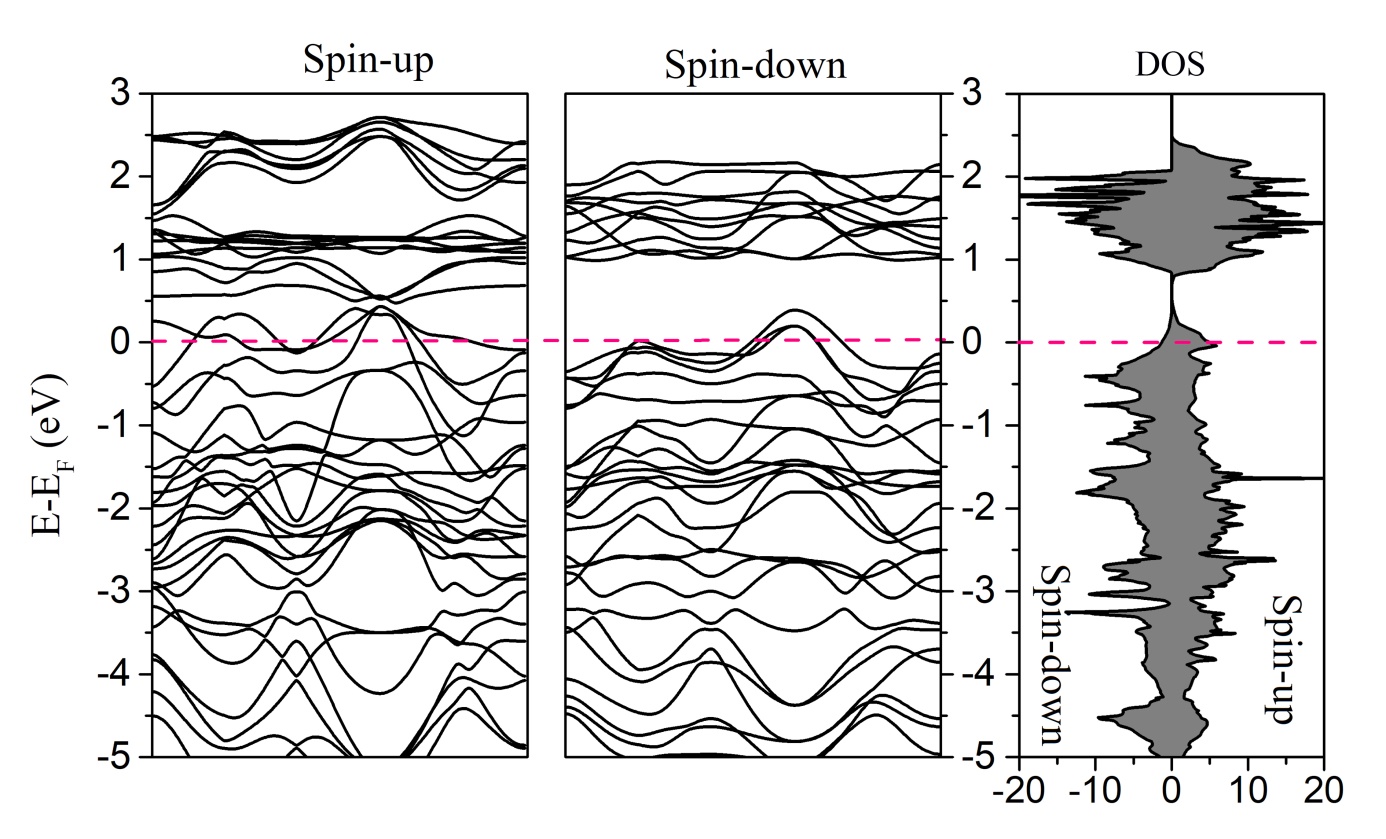


Fig. S2 The spin-polarized electronic band structure and the DOS of the Fe_B_-vac structure in the 14-atoms cell. The Fermi level was adjusted to zero and labeled as pink dashed line.
